# Direct and indirect effects of father-child attachment on academic burnout in college students

**DOI:** 10.3389/fpsyg.2024.1345590

**Published:** 2024-03-12

**Authors:** Zhenyun Zhang, Yuhua Wang, Huifen Wu, Yuqin Zhou, Cong Peng

**Affiliations:** ^1^School of Computer and Information Science, Hubei Engineering University, Xiaogan, China; ^2^School of Education and Psychology, Hubei Engineering University, Xiaogan, China; ^3^Research Center for Psychology and Behavior, Hubei Engineering University, Xiaogan, China; ^4^School of Foreign Languages, Hubei Engineering University, Xiaogan, China

**Keywords:** father-child attachment, academic burnout, core self-evaluation, sense of school belonging, multiple mediating effects

## Abstract

The study aims to investigate the multiple mediating roles of core self-evaluation and sense of school belonging in the relationship between father-child attachment and academic burnout in college students. A sample of 418 college students completed the father-child attachment scale, the scale of sense of school belonging, core self-evaluation scale, and academic burnout scale. After controlling for variables such as mother–child attachment, gender, age, and grade, the results showed: (1) father-child attachment was significantly and negatively correlated with academic burnout, and positively correlated with core self-evaluation and sense of school belonging; both core self-evaluation and sense of school belonging were significantly and negatively correlated with academic burnout. (2) The multiple mediating model of father-child attachment influencing academic burnout in college students was established. Both core self-evaluation and sense of school belonging played a partial mediating role between father-child attachment and academic burnout. The direct effect of father-child attachment on academic burnout accounts for 33.3% of the total effect. The indirect effects of core self-evaluation and sense of school belonging between father-child attachment and academic burnout account for 50.0 and 16.7% of the total effect, respectively. These findings identify the internal mechanisms through which father-child attachment affects academic burnout in college students from personal traits and interpersonal perspectives.

## Introduction

Academic burnout refers to a negative psychological and behavioral state in which students have low interest, lack of motivation, and tiredness in studying (Lian et al., 2014). A recent survey conducted among 3,648 samples found that academic burnout is prevalent among Chinese college students, with a prevalence rate of 38% (Peng et al., 2023). Academic burnout not only affects students’ academic performance (Gao, 2014), but also has negative impacts on their mental health (Shi et al., 2023). College students experiencing academic burnout are more prone to suffer from depression, anxiety, and internet addiction (Cheng and Lin, 2023; Nizam et al., 2023; Peng et al., 2023). Considering the total large number of Chinese college students in 2022, which has reached 46.55 million (data from the website of the Ministry of Education of the People’s Republic of China^1^), as well as the increasing cost of higher education, it is crucial to identify the factors influencing academic burnout among Chinese college students and explore intervention strategies to reduce it for the sake of students’ psychological well-being.

Factors influencing academic burnout among college students have been extensively studied. Previous research has mainly focused on two aspects: individual internal factors and external environmental factors. Individual internal factors include factors such as professional commitment (Lian et al., 2006), coping styles (Shi et al., 2023), academic anxiety (Gao, 2023), loneliness (Gu et al., 2023), self-esteem (Yu et al., 2023), achievement motivation (Atik and Çelik, 2021), personality (Lee et al., 2017; Kong et al., 2021), positive psychological capital (Wang et al., 2021), psychological resilience (Gong et al., 2023), self-efficacy (Chen et al., 2023), and core self-evaluation (Dortaj, 2021). External environmental factors include stress (Watson et al., 2008; Gong et al., 2023; Shi et al., 2023), social support (Ye et al., 2021; Fu, 2023), parental education anxiety (Kai et al., 2022), parental supervision (Affuso et al., 2017), and parent–child attachment (Zhang et al., 2019).

To date, the external factors influencing academic burnout among college students have received less attention from researchers compared to individual internal factors, with limited research focusing on key external factors such as family and school. Father-child attachment and mother–child attachment, as one of the most important proximal environmental factors, provide a solid foundation for individuals’ cognitive development and significantly influence their social adaptation (Yin et al., 2021). A few studies have confirmed the impact of parent–child attachment on learning burnout. However, relevant studies have mainly focused on the combined effects of parents or mothers’ individual impact on students’ academic achievement (He et al., 2022), yet little is known about the role of fathers in students’ academic performance (Whitney et al., 2018). The impact of father-child attachment on individual development is often overlooked or underestimated (Verschueren, 2019; Bureau et al., 2020). Research specifically examined the effects of father-child attachment on academic burnout among college students is still lacking.

Furthermore, the role of school environmental factors in alleviating academic burnout among college students should by no means be neglected. Schools belong to the microsystem of the social ecosystem, where college students invest a lot of time (Bronfenbrenner and Morris, 2006). Maslow’s original hierarchy of needs suggested a sense of belonging in the community is one of important human needs and a basis for self-realization (Hale et al., 2019). The sense of school belonging can be viewed as individuals’ sense of identification and affiliation with the school community. If students’ sense of school belonging is satisfied, it can promote their intrinsic learning motivation and have a positive effect on their learning behavior (Allen et al., 2022). Based on internal working model of attachment theory, individuals with good parent–child attachment can utilize stable internal working models in their daily academic interactions, which influence their expectations and adaptation in the school environment (Allen et al., 2004). Previous studies have addressed the significance of parent–child attachment in shaping college students’ sense of school belonging (Oldfield et al., 2018; Yin et al., 2021). Additionally, research indicated that parent–child attachment could impact students’ psychological well-being and social adaptation within their educational institution through the mediation of sense of school belonging (Shochet et al., 2008). However, these studies examined parent–child attachment as a combined variable of father-child and mother–child attachment. Therefore, the indirect impact of father-child attachment on academic burnout through sense of school belonging is undoubtedly worthy of further study.

In addition, solely focusing on the influence of external environmental factors such as family and school cannot fully reveal the influencing mechanism. It is necessary to comprehensively explore the impact of the individual internal factors. According to the self-model of attachment theory, the parent–child attachment relationship could profoundly influence an individual’s behavior and performance through the mental representations of the self (Bowlby, 1979). Core self-evaluation refers to an individual’s fundamental evaluation of their own abilities and worth, which consists of four important elements: self-esteem, locus of control, general self-efficacy, and neuroticism (Judge et al., 2003). Empirical research has substantiated the considerable influence fathers wield over various fundamental aspects of core self-evaluation in adolescents (Yang and Cai, 2010; Wu and Yaacob, 2017). Furthermore, prior studies have suggested that core self-evaluation stands as a robust individual predictor of burnout among undergraduate students (Leupold et al., 2020). However, whether core self-evaluation could be an important path for father-child attachment that affects academic burnout in college students remains unknown.

Finally, previous studies have either examined the effects of individual internal factors or external environmental factors on academic burnout, while less attention has been given to the simultaneous effects of both internal and external factors. According to the ecological systems theory (Bronfenbrenner and Morris, 2006), which suggests that the microsystems such as family and school environmental factors and individual factors collectively affect individual development. Based on findings and analysis above, this study intends to comprehensively examine the impact mechanisms of factors such as father-child attachment (the external family factor), sense of school being (external school factor), and core self-evaluation (the individual internal factor) on academic burnout among college students. This not only expands the research scope of the influencing factors of academic burnout but also reveals the unique role of fathers in preventing academic burnout among college students, and therefore has important implications for the intervention of academic burnout among college students in China.

### Father-child attachment and academic burnout in college students

Among the factors influencing academic burnout among college students, parent–child attachment plays an important role in the family context. Attachment refers to a stable and enduring emotional connection established between an individual and primary caregivers (i.e., mothers, fathers) during early life (Groh et al., 2016). Based on attachment theory, forming secure attachment relationships with important others can increase individuals’ internal resources, which in turn help them cope with life challenges and failures (Bowlby, 1969). Empirical studies have shown that high-quality parent–child attachment significantly improves children’s social adaptation, such as interpersonal, emotional, and learning adaptation (Yin et al., 2021). However, existing studies often investigate the effects of mother–child attachment or the combined effects of mother–child attachment and father-child attachment on academic burnout (i.e., Zhang et al., 2019; Iuga et al., 2023). By combining father-child attachment and mother–child attachment, the unique role of fathers may be overlooked. Furthermore, most existing research has focused on the impact of father-child attachment on academic outcomes among children and adolescents (Flouri and Buchanan, 2004; Martin et al., 2010; Whitney et al., 2018), with limited attention given to its effects on college students’ academic performance. Research suggests that in late adolescence, the influence of fathers on their children’s academic achievement is comparable to that of mothers (Vasquez et al., 2016). A meta-analysis of 34 studies found that fathers played a unique role in nurturing their children, and there is a significant correlation between fathers’ involvement and children’s outcome variables such as academic performance (Jeynes, 2016).

According to the Modeling Hypothesis, a person learns and imitates attitudes and behaviors from significant others who have an important influence on them (Thorn and Gilbert, 1998), making fathers a crucial influential role. Children often consciously or unconsciously mimic their fathers’ behavior and internalize their attitudes and values toward things. Fathers’ pursuit of higher goals and expectations for their children’s academic achievement have a strong motivating effect. Fathers’ care and attention to their children’s academic endeavors can instill confidence and motivation, helping them actively engage in studying. Besides, while studying the impact of fathers on children’s academic adaptation, it is necessary to take cultural differences into consideration, because the quality of father-child relationship varies in different cultural backgrounds and its impact on individual development exhibits different characteristics (Lamb, 2010). In traditional Chinese culture, the popular parenting style of *yan-fu-ci-mu* (strict father and kind mother) indicates that the father is primarily characterized as a stern educator and responsible for shaping children’s future development (Peng et al., 2022). However, there is a lack of research specifically focused on the impact of father-child attachment on academic burnout among Chinese college students. Therefore, it is necessary to explore the effects and mechanisms of father-child attachment on Chinese college students’ academic burnout. This study proposes Hypothesis 1: Father-child attachment has a significant effect on academic burnout in Chinese college students.

### The mediating effect of core self-evaluation

Core self-evaluation (CSE) refers to an individual’s fundamental evaluation of their own abilities and worth (Judge et al., 2003). CSE has been found to significantly impact students’ academic burnout in multiple studies. College students with higher levels of CSE reported lower levels of burnout (Lian et al., 2014). Students with positive core self-evaluations are more likely to proactively deal with challenging situations, which can increase their burnout (Paloș, 2023). Leupold et al. (2020) indicated that CSE is a strong individual predictor of burnout among undergraduate students. Based on the approach-avoidance framework (Ferris et al., 2011), individuals with high CSE are more sensitive to positive stimuli and less sensitive to negative stimuli, exhibiting strong approach tendencies and weak avoidance tendencies. Therefore, individuals with high CSE demonstrate more resilience and determination to improve their academic situations.

Attachment theory suggests that parent–child attachment is an emotional connection established between an individual and significant others that has a sustained impact on their psychological and behavioral development. Individuals with high levels of attachment tend to believe that they are lovable, valuable, and capable of coping with life challenges due to receiving good care, which enhances their self-esteem (Peng et al., 2022). CSE consists of four important elements: self-esteem, locus of control, general self-efficacy, and neuroticism (Judge et al., 2003). Studies have shown that fathers have significant effects on the four basic traits of adolescents’ CSE, such as self-esteem (Peng et al., 2022), general self-efficacy (Keshavarz and Mounts, 2017), neuroticism (Yang and Cai, 2010), and locus of control (Lancaster and Richmond, 2012). However, there is little research that examines the impact of father-child attachment on college students’ core self-evaluation as a whole.

Meanwhile, studies have shown that parent–child attachment can affect children’s psychological and social adaptation through their CSE (He et al., 2023). It is not difficult to understand that high-level parent–child attachment can effectively increase an individual’s internal resources and help them recover faster from academic pressure and setbacks. Aspelmeier and Kerns (2003) analyzed the relationship between attachment and academic exploration among college students and found that the higher an individual’s attachment level, the less anxious their academic performance is, and the more positive attitude they have toward their studies. Parent–child attachment among college students affects their learning burnout by the self-model of attachment, which refers to an individual’s evaluation of self-worth (Yang et al., 2013). Therefore, it’s reasonable to assume that high core self-evaluation can be the result of a good father-child attachment relationship, that is, a stable father-child attachment relationship helps to cultivate a healthy core self-evaluation in individuals and ultimately affects the level of academic burnout. We propose Hypothesis 2: Core self-evaluation plays a mediating role between father-child attachment and academic burnout.

### The mediating effect of sense of school belonging

Sense of school belonging is typically defined as the degree to which students feel accepted, respected, and supported in the school environment (Allen et al., 2018). Sense of school belonging can mediate the relationship between father-child attachment and academic burnout. First, based on internal working model of attachment theory, when children form secure internal working models through positive parent–child interactions, perceiving stability, trust, and availability from their attachment figures, they develop the skills and strategies necessary for healthy interpersonal relationships and gain acceptance and recognition from teachers and peers (Pianta, 1999). The presence of a supportive teacher-student relationship and a secure attachment to peers can serve as protective factors in situations of learning challenges (Prino et al., 2016; Zou et al., 2020). These positive interpersonal relationships enable students to integrate into the university environment more quickly, thereby developing a sense of school belonging. Limited studies have shown that parent–child attachment is an important factor influencing college students’ sense of school belonging (Yin et al., 2021), and parental disengagement and adolescent school attachment were closely related (Shochet et al., 2007; Oldfield et al., 2016). However, these studies examined parent–child attachment as a combined variable of father-child and mother–child attachment. The impact of father-child attachment remains unknown.

Research has shown that parent–child attachment can affect students’ mental health and social adaptation in school through the mediation of sense of school belonging (Shochet et al., 2008). On the one hand, when college students have a high sense of school belonging, it can help with academic performance and improve attendance rates (Lam et al., 2015). On the contrary, college students with a low sense of belonging to school will show more learning fatigue, lack interest in study or learning motivation (Aker and Şahin, 2022). On the other hand, the lack of school belonging may lead to emotional problems for students, such as anxiety, loneliness, and depression (Shochet et al., 2008; Oldfield et al., 2018). These negative emotions can impair students’ learning motivation and efficiency, leading to the occurrence of learning burnout. Therefore, it can be considered that sense of school belonging may play a mediating role between father-child attachment and academic burnout. This study proposes Hypothesis 3: Father-child attachment may affect college students’ academic burnout through the mediation of their sense of school belonging.

### Current research

**Figure 1:**
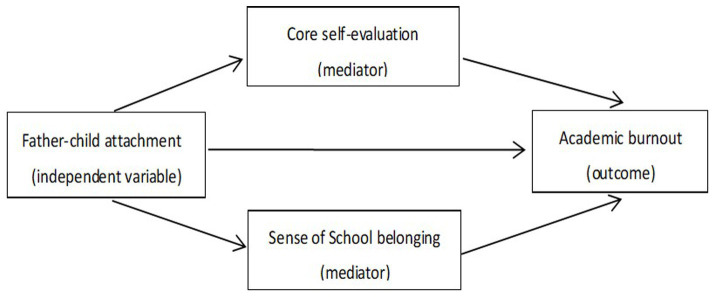
The proposed multiple mediation model.

On the basis of attachment theory and the ecological systems theory, this study constructed a multiple mediation model, taking father-child attachment as an independent variable, academic burnout as a dependent variable, both core self-evaluation and sense of school belonging as mediating variables. We propose the hypotheses that father-child attachment negatively affects academic burnout, and it significantly positively affects core self-evaluation and sense of school belonging. Core self-evaluation and school belonging may serve as parallel mediators in the relationship between father-child attachment and academic burnout among Chinese college students (see Figure 1).

## Materials and methods

### Participants

The participants were 418 students recruited from three universities of Hubei Province, China. A group test was carried out. A total of 453 anonymous questionnaires were distributed and 418 valid questionnaires were collected, with an effective rate of 92.3%. The age of the subjects ranged from 17 to 25 years old, with an average age of 19.29 years (SD = 1.36 years). There were 143 male students (34.2%) and 275 female students (65.8%). The participants consisted of 134 (32.1%) in Grade One, 165 (35.9%) in Grade Two, 101 (24.2%) in Grade Three, and 18 (4.3%) in Grade Four.

### Measures

#### Father-child and mother–child attachment scale

The father-child attachment and mother–child attachment subscales in the Chinese version of the Inventory of Parent and Peer Attachment (IPPA) (Armsden and Greenberg, 1987) revised by Bao and Xu (2006) were used for measurement. The two subscales contained 25 questions (i.e., “My Dad respects my feeling.”, “I tell my mother about my problems and troubles.”), both including three dimensions of trust, communication, and alienation. All the items were presented in the form of a 5-point Likert-type scale (1 = *almost never*, 5 = *almost always*). Participants with higher total scores suggested that they had higher level of attachment to their fathers or mothers. In this study, the Cronbach’s α for two scales were 0.869 and 0.885, respectively.

#### Core self-evaluation scale

The Scale of Core Self-evaluations (Judge et al., 2003) was adopted, which had 12 items, such as “I am confident I get the success I deserve in life.” The 5-point Likert scale ranging from “strongly disagreeable” to “strongly agreeable” was used, with higher total scale indicating higher levels of core self-evaluation. In this study, the Cronbach’s α of the scale is 0.805.

#### Psychological sense of school membership

A revised Chinese version of Scale of Psychological sense of school membership (Goodenow, 1993) was applied to test the sense of school belonging, which had 18 items, such as “I feel like a part of my school.” The 6-point Likert scale ranging from “Not at all true” to “Completely true” was adopted, where higher scores indicate higher degree of sense of school belonging. In this study, the Cronbach’s α of the scale is 0.884.

#### Academic burnout scale for college students

The Academic Burnout Scale for College students developed by Lian et al. (2006) was adopted, which included 3 dimensions such as physical and mental exhaustion, improper behavior, and low achievement. The scale contained 20 items (i.e., “I want to study, but I find it very boring,” “I seldom study after class”), which was presented in the form of a 5-point Likert scale ranging from “strongly disagreeable” to “strongly agreeable” was used, with higher total scale indicating higher levels of academic burnout. In this study, the Cronbach’s α of the scale is 0.870.

#### Control variables

In addition to the main variables of interest, we controlled for mother–child attachment to investigate the unique impact of father-child attachment. The impact of father-child attachment on children’s development may be influenced by the level of mother–child attachment. Previous studies have found that father-child attachment and mother–child attachment have a compensatory effect on the development of children, with one parent alleviating the negative impact of the other (Verschueren and Marcoen, 1999; Zhao, 2012). Therefore, mother–child attachment in this study was controlled in order to analysis the independent effect of father-child attachment. Gender, age, and grade were also controlled to avoid these variables’ influence. Research showed that the associations between father-child attachment and children’s developmental outcomes will vary with age, gender, and development stage (Verschueren, 2019).

#### Procedure

Informed consent to participate in this study was obtained from all participants before the data collection. Participants were informed that their participation was voluntary and they could terminate the participation at any time. The Ethical approval was granted by the Ethical Committee at Hubei Engineering University. Moreover, teachers from each university were recruited and trained as research assistants to completed the distribution and recycling of questionnaires, and answered the participants’ any questions immediately. The participants were given approximately 30 min to complete the survey.

#### Data analysis

SPSS version 23.0 was used for descriptive statistics and correlation analysis. The Pearson’s correlation analysis was applied to analyze the correlation coefficient between variables. Then Model 4 in PROCESS macro program for SPSS (Hayes, 2012) was conducted to investigate the multiple mediating effects of core self-evaluation and sense of school belonging between father-child attachment and academic burnout among college students. The 95% bias-corrected bootstrap confidence interval was selected based on 5,000 bootstrap samples to estimate the statistical significance of the multiple mediating effect.

## Common method bias tests

As the data in this study comes from participants’ self-reports, the deviation caused by the same data collection method might occur. To exclude this possibility, Harman’s single-factor test method was applied to detect the common-method bias. According to the results of non-rotating principal component factor analysis, 15 common factors with eigenvalues greater than 1 were extracted, and the variation explained by the first factor was 21.65% (less than the 40% of standard threshold), indicating that there was no serious common-method bias in this study (Podsakoff et al., 2003).

## Results

### Preliminary analysis

The mean, standard deviation, and correlation coefficient of father-child attachment, mother–child attachment, core self-evaluation, sense of school belonging, and academic burnout are shown in Table 1. The results showed that father-child attachment was significantly negatively correlated with academic burnout (*p* < 0.01), and positively correlated with core self-evaluation and sense of school belonging (*p* < 0.01); core self-evaluation was positively correlated with sense of school belonging (*p* < 0.01), and negatively correlated with academic burnout (*p* < 0.01); sense of school belonging was negatively correlated with academic burnout (*p* < 0.01).

**Table 1:** The correlation of the main study variables (*N* = 418).

Variables	*M* ± SD	1	2	3	4	5	6	
1 FCA	3.45 ± 0.71	1						
2 MCA	3.65 ± 0.72	0.58**	1					
3 CSE	3.55 ± 0.54	0.38**	0.40**	1				
4 SSB	4.01 ± 0.70	0.35**	0.40**	0.50**	1			
5 AB	2.90 ± 0.52	−0.37**	−0.35**	−0.40**	−0.61**	1		
6 Gender	0.65 ± 0.48	−0.02	0.07	0.16**	−0.04	−0.04	1	
7 Age	19.18 ± 1.37	0.06	−0.02	−0.14**	−0.08	0.02	−0.24**	1

*p < 0.05; **p < 0.01; Gender was a binary variable (0 = male; 1 = female); FCA, father-child attachment; MCA, mother–child attachment; CSE, core self-evaluation; SSB, sense of school belonging; AB, academic burnout.

### Testing for multiple mediating effect

**Figure 2:**
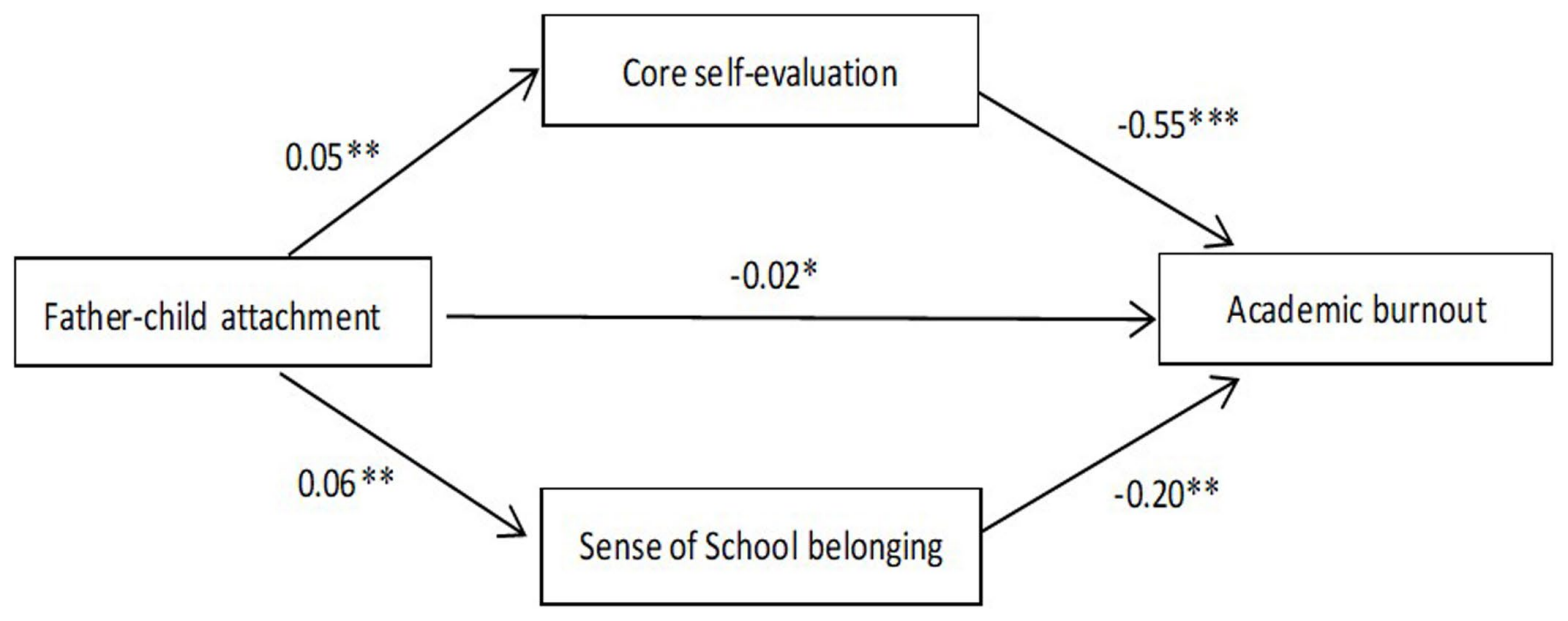
The multiple mediating effects of core self-evaluation and sense of school belonging.

It can be seen from Table 1 that father-child attachment, core self-evaluation, sense of school belonging and academic burnout are significantly correlated with each other. In this study, the 95% bias-corrected bootstrap confidence interval was selected based on 5,000 bootstrap samples to estimate the statistical significance of the multiple mediating effect, and Model 4 (a mediation model) in SPSS macro compiled by Hayes (2012) was used to test it. Under the condition of controlling for mother–child attachment, gender, age, and grade, the results of multiple mediation regression analysis (see Table 2; Figure 2) showed that the independent variable father-child attachment could significantly affect core self-evaluation (*B* = 0.05, *t* = 3.71, *p* < 0.01); father-child attachment significantly influenced sense of school belonging (*B* = 0.06, *t* = 3.26, *p* < 0.01); core self-evaluation (*B* = −0.55, *t* = −11.32, *p* < 0.001), sense of school belonging (*B* = −0.20, *t* = −5.94, *p* < 0.001) and father-child attachment (*B* = −0.02, *t* = −1.99, *p* < 0.05) significantly affected academic burnout.

**Table 2:** Testing for the multiple mediating effects.

Regression equation (*N* = 418)		Fitting indicators			Coefficient significance	
Outcomes	Predictors	*R*	*R^2^*	*F*	*β*	*t*
Core self-evaluation		0.42	0.18	15.83***		
	Gender				−0.03	−0.63
	Age				0.02	0.81
	Grade				−0.01	0.81
	Mother–child attachment				0.06	4.30***
	Father-child attachment				0.05	3.71**
Sense of school belonging		0.44	0.19	17.77***		
	Gender				−0.11	−1.62*
	Age				−0.04	−1.06
	Grade				−0.03	−1.06
	Mother–child attachment				0.09	5.06***
	Father-child attachment				0.06	3.26**
Academic burnout		0.75	0.56	67.21***		
	Gender				−0.07	−1.68**
	Age				0.01	−0.50
	Grade				0.04	1.16
	Mother–child attachment				0.001	0.11
	Core self-evaluation				−0.55	−11.32***
	Sense of school belonging				−0.20	−5.94***
	Father-child attachment				−0.02	−1.99*

*p < 0.05, **p < 0.01, ***p < 0.001.

In addition, Table 3 shows the mediating effects of father-child attachment on academic burnout. The direct effect of father-child attachment on academic burnout is-0.02 (*t* = −1.99, *p* < 0.05, LLCI = −0.04, ULCI = −0.002), accounting for 33.3% of the total effect (−0.06), and the total indirect effect of core self-evaluation and sense of school belonging between father-child attachment and academic burnout is −0.04, 95% [−0.07, −0.02], accounting for 66.7% of the total effect (−0.06). The indirect effect of core self-evaluation between father-child attachment and academic burnout is −0.03, 95% [−0.05, −0.01], accounting for 50.0% of the total effect (−0.06). The indirect effect of sense of school belonging between father-child attachment and academic burnout is-0.01, 95% [−0.03, −0.001], accounting for 16.7% of the total effect (−0.06). The bootstrap 95% confidence interval of the mediating effects of both core self-evaluation and sense of school belonging did not contain 0 value. All standardized effects are statistically significant (see Table 3). This indicates that core self-evaluation and sense of school belonging have a multiple mediating effect between father-child attachment and academic burnout.

**Table 3:** The estimates of total, direct and indirect effects of the model.

Indirect effect		Boot SE	Boot LLCI	Boot ULCI	Relative mediation effect
Total indirect effect	−0.04	0.02	−0.09	−0.03	66.7%
FCA → CSE → Academic Burnout	−0.03	0.01	−0.09	−0.05	50.0%
FCA → SSB → Academic Burnout	−0.01	0.003	−0.01	−0.001	16.7%

FCA, father-child attachment; CSE, core self-evaluation; SSB, sense of school belonging.

## Discussion

After controlling for the variables of mother–child attachment, gender, age, and grade, this study found that the level of father-child attachment significantly and negatively influenced college students’ academic burnout. This supports Hypothesis 1 and is consistent with previous research that highlights the significant role of fathers in children’s academic performance (Coley et al., 2011; Affuso et al., 2017; Whitney et al., 2018). Previous studies have found that fathers in Chinese families are highly involved in their children’s education and place great importance on their academic performance (Xu, 2016). According to the Father Activation Theory (Paquette and Bigras, 2010), positive father-child relationships can stimulate children’s interest in exploration and encourage them to face challenges in various aspects of their lives, including learning and interpersonal relationships. Fathers often possess personality traits such as confidence, initiative, independence, and the ability to confront challenges and overcome difficulties. Long-term positive interactions and guidance from fathers can help students effectively cope with learning difficulties, establish reasonable academic goals, and actively engage in learning to avoid academic burnout. On the contrary, because of lacking of father’s activation, individuals with a estranged father-child relationship may dare not to explore one’s own goals in the loose environment of Chinese universities. Chinese colleges and universities still follows the pattern which is strict at entrance but casual to out (Feng, 2009), which makes the young realize that obtaining a degree of higher education is an easy matter. Therefore, lack of learning motivation and academic self-efficacy making them more prone to academic burnout at colleges and universities.

### The mediating role of core self-evaluation

Moving on to the mediating effects, this study found that father-child attachment significantly affected college students’ core self-evaluation. Core self-evaluation partially mediates the relationship between father-child attachment and academic burnout, supporting Hypothesis 2. According to attachment theory’s internal working model (Bowlby, 1979), the internal working model formed through interactions with significant others includes a critical part related to core self-evaluation. When the father-child dyad involves positive interactions, fathers can provide emotional support and enhance their children’s self-worth and self-acceptance, promoting positive self-esteem development (Peng et al., 2022). Good father-child attachment have a negative effect on the level of neuroticism among adolescents (Yang and Cai, 2010). Father closeness significantly influenced their children’s self-efficacy (Wu and Yaacob, 2017), and individuals from father-absent homes were found to have a higher external locus of control than those from father-present homes (Lancaster and Richmond, 2012). Additionally, based on the approach-avoidance framework (Leupold et al., 2020), individuals with high core self-evaluation are less likely to internalize harsh critiques of their academic coursework and are more likely to utilize criticism as an opportunity for growth. On the other hand, individuals with low core self-evaluation may view the same feedback as a demotivating personal affront. Studies have shown that core self-evaluation is an important protective factor for individuals’ academic development (Lian et al., 2014). Individuals with high core self-evaluation are more adept at proactively coping with academic stress (Li et al., 2019). Conversely, individuals with low core self-evaluation are more likely to exhibit academic aversion, procrastination, and academic burnout (Lian et al., 2014; Rajapakshe, 2018). Based on these findings, it can be understood that core self-evaluation mediates the relationship between father-child attachment and academic burnout.

### The mediating role of sense of school belonging

Furthermore, this study found that father-child attachment significantly affected college students’ sense of school belonging, and sense of school belonging partially mediates the relationship between father-child attachment and academic burnout, supporting Hypothesis 3. This finding is in line with previous research on the relationship between parent–child attachment and school belonging (Yin et al., 2021). In traditional Chinese families, fathers serve as role models, and children often mimic their fathers’ behaviors and ways of handling things, forming a set of patterns during interactions with their fathers. Research indicated that father-child relationships focus on the social sphere and are closely related to the individual’s subsequent relationships (Paquette, 2004). Based on attachment theory, individuals construct internal working models about themselves, others, and interpersonal relationships from early attachment-relevant experiences with parents (Bowlby, 1979). The internal working models established through early social interactions naturally extends to an individual’s other interpersonal relationships, including peer relationships and students-teacher relationships, and indirectly affects their adaptation (Ma and Huebner, 2008). If college students learn good interpersonal communication skills from fathers, they will experience positive effects in their school life and learning, and establish good relationships with teachers and peers (Cabrera et al., 2011; Tan et al., 2023), ultimately forming a sense of school belonging.

Sense of school belonging refers to students’ sense of identification and attachment to the school community, which plays an important role in their motivation and emotional well-being, helping them cope with academic stress. According to Maslow’s theory of self-fulfillment, the satisfaction of an individual’s sense of belonging to their group is an important psychological need and a fundamental basis for pursuing self-actualization (Li and Singh, 2023). Students with a high sense of school belonging tend to demonstrate better metacognition, time management skills, and engage more in cooperative learning with peers (Won et al., 2018). Conversely, students with a low sense of school belonging exhibit maladaptive learning behaviors, lack interest or motivation in learning, exhibit high levels of procrastination, experience poor peer relationships, and demonstrate poor self-regulation skills (Kennedy and Tuckman, 2013; Oldfield et al., 2016). Additionally, students with low school belonging are more likely to experience negative emotions in school (Oldfield et al., 2018), which can further impact their academic performance. When college students establish a good father-child relationship with mutual trust and smooth communication, they are more likely to feel accepted, respected, and belonged at college. This feeling enhances students’ identification and sense of school belonging, thereby improving their motivation and academic achievement. Therefore, higher father-child attachment can enhance students’ sense of school belonging, thereby reducing their level of academic burnout.

### The multiple mediating effects

The analysis of multiple mediation models found that in addition to having a direct effect on learning burnout, father-child attachment can also have a parallel mediation effect through core self-evaluation and sense of school belonging. Father-child attachment can affect an individual’s self-evaluation and the formation of sense of school belonging, thereby affecting the development of academic burnout. Therefore, individuals with high level of father-child attachment, positive self-evaluation, and good sense of school belonging are likely to have high autonomy and learning motivation, thereby reducing the occurrence of learning burnout. This is consistent with the ecological systems theory, the family is the most important proximal environment that affects individual development, and micro systems such as schools and individual characteristics also have a significant impact on individual development (Bronfenbrenner and Morris, 2006). Family environment variables do not affect students’ development through a single direct path. They can indirectly affect students’ academic development through multiple paths such as individual psychological characteristics or students’ psychological states in the new environment.

### Limitations and implications

While this study provides important insights into the relationships between father-child attachment, core self-evaluation, sense of school belonging, and academic burnout, there remain some limitations in this study. First, as a cross-sectional study, it cannot establish causal relationships between the variables. Future longitudinal studies can provide a clearer understanding of the temporal nature of these relationships. Second, all the data used in this study relied on self-reported questionnaires, which may be subject to social desirability bias and self-defense mechanisms. Future studies could incorporate multiple perspectives, including parents, peers, and teachers’ evaluations, to obtain a more comprehensive understanding of the variables. Additionally, it is important to acknowledge that college students develop significant attachment relationships with peers, seeking emotional support from their peers to fulfill their need for belonging and intimacy and cope with academic failure. Future research can adopt an integrated perspective that considers family, individual, and interpersonal factors within the micro-environmental systems of college students’ academic development. Lastly, the sample were recruited from a specific province in central China, so caution should be required when interpreting the findings within different regions or outside of China.

Despite these limitations, the findings of this study have the following implications. When intervening with college students experiencing academic burnout, it is crucial for college administrators and practitioners to consider father-child attachment as a key factor related to students’ academic performance. Interventions can focus on improving father-child relationships, promoting effective communication, and enhancing mutual trust between fathers and children. Fathers can get involved in guiding the grown-up children to set academic goals they want to pursue, avoid negative evaluations of children experiencing academic setbacks, encourage them to overcome learning difficulties, enhance their self-esteem and academic self-efficacy, and increase their confidence in dealing with academic challenges. Additionally, fathers should pay special attention to their children’s integration into the university environment, encourage them to foster and sustain harmonious relationships with classmates, promote cooperation with peers, and provide positive guidance in establishing positive interactions with teachers to enhance their sense of school belonging, ultimately improving their learning motivation and academic achievement.

## Conclusion

This study has explored the relationships between father-child attachment, core self-evaluation, sense of school belonging, and academic burnout among college students. The findings suggest that father-child attachment has direct and indirect effects on academic burnout, with core self-evaluation and sense of school belonging serving as parallel mediators. These findings contribute to our understanding of the internal mechanisms through which father-child attachment affects academic burnout in college students and highlight the importance of the father’s role in prevention and reduction of academic burnout among Chinese college students.

## Data availability statement

The original contributions presented in the study are included in the article/supplementary material, further inquiries can be directed to the corresponding author.

## Ethics statement

The studies involving humans were approved by the Ethical Committee at Hubei Engineering University. The studies were conducted in accordance with the local legislation and institutional requirements. The participants provided their written informed consent to participate in this study.

## Author contributions

ZZ: Conceptualization, Data curation, Formal analysis, Investigation, Methodology, Resources, Software, Writing – original draft, Writing – review & editing. YW: Data curation, Formal analysis, Investigation, Methodology, Writing – review & editing. HW: Data curation, Formal analysis, Investigation, Resources, Software, Writing – review & editing. YZ: Investigation, Resources, Validation, Writing – review & editing. CP: Conceptualization, Funding acquisition, Investigation, Methodology, Project administration, Supervision, Validation, Writing – original draft, Writing – review & editing, Data curation.

## Footnotes

^1^http://www.moe.gov.cn/fbh/live/2023/55167
